# Effects of college students’ mindfulness on depression symptoms during the epidemic prevention and control period: The mediating effect of psychological resilience

**DOI:** 10.3389/fpsyt.2022.991449

**Published:** 2023-01-04

**Authors:** Yanfei Jiang, Zhiyu Yi, Youjuan Yao, Yanbing Hu, Feilin Li, Huizhen Ma

**Affiliations:** ^1^Key Laboratory of Behavior and Mental Health of Gansu, Department of Psychology, Northwest Normal University, Lanzhou, China; ^2^Department of Psychology, Northwest Normal University, Lanzhou, China; ^3^Institute of Linguistics, Shanghai International Studies University, Shanghai, China

**Keywords:** depression symptoms, mindfulness, psychological resilience, COVID-19, mental health

## Abstract

Depression symptoms significantly impact college students’ mental health, particularly during the “closed management” period during the spread of COVID-19. Exploring the mechanism that affects college students’ depression symptoms can help alleviate the impact of closed management policies on individual mental health and improve their mental health level. The onset of the COVID-19 pandemic resulted in the normalization of epidemic prevention and control in China and the implementation of the dynamic zero-COVID policy. This study used the Five-Factor Mindfulness Questionnaire—Short Form, Psychological Resilience Scale, and Beck Depression Scale to investigate the mindfulness, psychological resilience, and depression symptoms of 1,062 students under closed management conditions at Northwest Normal University. The mindfulness, psychological resilience, and depression status of students in closed management were investigated using an online questionnaire survey. Eight hundred and ten college students (*M*_*age*_ = 20.43, *SD* = 1.67, range = 17-30) were selected to test the model using the structural equation model and bootstrap method. The results showed that the gender differences in mindfulness and psychological resilience were not significant. Gender differences in depression symptoms were significant, and depression symptoms in men were significantly higher than in women. Grade differences in resilience, mindfulness, and depression levels were not significant. Thus, psychological resilience is negatively associated with depressive symptoms. Psychological resilience plays a mediating role between mindfulness and depressive symptoms. This study provides reference and inspiration for improving college students’ mental health under epidemic prevention and control circumstances.

## 1 Introduction

Since December 2019, the novel coronavirus disease 2019 (COVID-19) has become endemic worldwide. Due to the severity of its clinical symptoms and the widespread infections, COVID-19 has significantly damaged the global economy and human health (1). As China enters the normalization of epidemic prevention and control stage,^1^ China’s dynamic zero-COVID policy^2^ has become the general policy. While travel restrictions and closed public spaces policies have achieved considerable results (4), they have also significantly impacted the mental health of residents with limited living space. These residents’ mental health level is generally poor, exhibiting symptoms of psychological stress (5). Many colleges and universities have adopted closed management measures to curb the virus’s spread. This means that teachers and students are prohibited from moving across campuses; teachers are restricted to their homes, while students are restricted to their dormitories. Under closed management, teaching is conducted online; libraries, study rooms, stadiums, and other public places are temporarily closed; hall meals are canceled; meals are taken at staggered times in the campus canteen; and express delivery and takeaways are suspended. The epidemic has led to changes in lifestyle, isolation at home or in schools, and concerns about unmet basic living needs (6, 7). These pose several challenges to college students (8), inducing high levels of depression (9). Meta-analysis showed that during the outbreak of COVID-19, the depression score of college students was significantly higher than that of other groups; the rate of severe depression particularly, was approximately 20% higher than before the epidemic (10). This finding suggests that more attention should be paid to the mental health needs of college students. The change in college students’ lifestyles under closed management epitomizes the change experienced in all Chinese residents’ lifestyles under the epidemic prevention policy. Exploring college students’ depression status and influence mechanisms during the epidemic prevention and control period is also relevant to understanding citizens’ mental health levels under the same situation. This information can help in formulating targeted intervention measures for various populations.

Mindfulness is a protective factor helping individuals recover from adversity and pressure (11). It is of particular significance to college students facing the external pressures of epidemic prevention and control. Mindfulness is a conscious and non-judgmental awareness focusing on the present moment (12). It is an individual’s ability to maintain attention and awareness of the present moment and is often regarded as a trait-like psychological state (13). The mindfulness reperceiving model can explain the mindfulness mechanism promoting mental health. People who consciously adopt an objective and non-judgmental attitude can better manage their present circumstances and may even recognize the positive meaning underlying adverse events, leading to successful self-regulation (14). Additionally, mindfulness encourages individuals to search for meaning and more actively manage negative events (15). Xu et al. (16) demonstrated that mindfulness is an internal resource that helps injured individuals adjust and improve their psychological resilience. In the epidemic environment, college students’ mindfulness is the most protective factor related to depression and stress perception. High-level mindfulness can reduce pain and depression symptoms caused by the epidemic and significantly improve individual tolerance of pressure (17).

Mindfulness is directly and negatively associated with an individual’s depression symptoms (18, 19). Individuals with higher levels of mindfulness usually exhibit better mental health and lower negative emotions. Such individuals are also effective at focusing on the present; they can better distinguish emotions and adopt a more objective perspective to recognize themselves and various life events through self-regulation (20, 21). Individuals with lower mindfulness levels are more likely to experience depression when facing stressful events (22). Empirical studies have shown that mindfulness interventions significantly ameliorate clinical groups’ anxiety and depression levels (13, 23). Furthermore, for non-clinical individuals, mindfulness can increase positive emotions, and mitigate negative ones (24).

Psychological resilience, which is also an important protective factor for mental health (25), refers to an individual’s ability to overcome adversity and restore normality; it is a relatively stable and universal personality trait (26, 27). Some scholars also believe that psychological resilience is a dynamic process for individuals to adapt well to dangerous environments (28). The framework of resilience in action posits that psychological resilience, as a type of innate potential, promotes the development of varied positive personal characteristics. These might include self-efficacy and cooperation, which constitute the internal resources necessary for individuals to manage stress in the future (29). Kumpfer’s resilience framework explains the mechanism of psychological resilience. When individuals face risk, psychological resilience facilitates active emotional and cognitive regulation, which can better integrate individuals’ internal and external resources to cope with stress (30). Rutter’s (31) psychoelastic development model emphasizes that psychological resilience is not targeted at reducing the individual’s contact with risk factors. Rather, it aims to develop the individual’s internal strength, dampening the impact of crisis events, and blocking the negative reactions precipitated by risk factors to enhance the individual’s ability to cope with stress and frustration. When individuals experience setbacks, those with high psychological resilience exhibit less psychological distress, fewer psychopathological symptoms, and better mental health than those with low psychological resilience (32, 33). In the epidemic context, psychological resilience is crucial to effectively cope with difficulties, uncertainties, and changes that affect the individual’s perception of pressure and prevent maladaptive behaviors (34, 35).

Mindfulness and psychological resilience are closely related as protective factors of mental health. Research has shown a significant positive correlation between individuals’ mindfulness traits and psychological resilience. Mindfulness traits are the internal psychological resources that improve psychological resilience. Mindfulness training also significantly improves practitioners’ cognitive function and psychological resilience (36, 37). Mindfulness training emphasizes the unconditional acceptance of emotion and cognition, which is conducive to improving the individual’s sense of cohesion and psychological resilience. Additionally, psychological resilience is significantly negatively correlated with negative psychological experiences such as depression (38). Individuals with low psychological resilience exhibit more severe depressive symptoms (39, 40), while individuals with high psychological resilience are more effective at utilizing positive emotions to resist the impact of depression symptoms. These individuals are also more likely to recover from negative emotions when facing pressure, learn from them, and exhibit an improved level of mental health after stressful events (41, 42). The present study hypothesizes that psychological resilience is the explanatory mechanism of mindfulness on depression symptoms. Specifically, mindfulness, through the acceptance of emotion and cognition, uses emotion regulation strategies (such as changing the way of perceiving stress), enhancing psychological resilience, reducing depression levels, and promoting better mental health.

The research on mindfulness, psychological resilience, and depression symptoms is still controversial. Some studies point out that psychological resilience bridges mindfulness and mental health (43, 44). However, other studies have shown inconsistent results, such as no linear relationship between psychological resilience and depression symptoms (45). These differences may be because most existing studies are conducted in particular groups, such as chronic gastritis patients, patients in the recovery period of depression, and women undergoing pregnancy termination for fetal abnormality. Moreover, the demographic characteristics of the research group also impact the results. Early studies on the COVID-19 epidemic indicated that women had high levels of mindfulness and depression symptoms and low levels of psychological resilience (46–48). In addition, the mental health level of older adults was higher than that of young individuals (49). However, with the continuation of the epidemic and an increase in citizens’ understanding of it, the impact of demographic variables on mental health gradually decreased (7). Therefore, whether the mindfulness, psychological resilience, and depression symptoms models can be extended to more representative groups (such as college students) and tested in the current epidemic environment remains to be seen. Given the context and the influence of the COVID-19 pandemic, the characteristics of depression symptoms, mindfulness, and psychological resilience have undergone some changes. Specifically, mindfulness and psychological resilience have had a positive impact on the individual’s ability to cope with the COVID-19 epidemic, becoming a topic of interest to researchers and other persons. Concurrently, false information, negative emotions, and lifestyle changes have a continuous negative impact on protective factors such as psychological resilience. The epidemic has aggravated individuals’ depressive symptoms. The depression diagnosis rate during the epidemic has increased significantly compared to before the epidemic (10, 17, 34). In summary, we selected ordinary college students under closed management conditions as the research object to establish a mediation model. In the context of the large-scale public health event of COVID-19, we explored the impact of mindfulness and psychological resilience on depression symptoms in a more representative sample group. This is of theoretical and practical significance as it can help us to engage with the challenges of the COVID-19 epidemic in a more targeted manner.

This study proposes the following hypotheses: Hypothesis 1: There are significant differences in depressive symptoms, psychological resilience, and mindfulness between genders and grades. Hypothesis 2: A significant negative correlation exists between college students’ mindfulness and depression symptoms during the epidemic prevention and control period; a significant positive correlation exists between mindfulness and psychological resilience; a significant negative correlation exists between psychological resilience and depression symptoms. Hypothesis 3: In the context of epidemic prevention and control, psychological resilience mediates the effect of mindfulness on depression symptoms.

## 2 Materials and methods

### 2.1 Participants

In this study, Questionnaire Star (a widely used data collection platform in China) was used to investigate college students’ mindfulness, psychological resilience, and depression symptom levels during the epidemic prevention and control period. The data were collected in April 2022 (1 month after the initiation of closed management). All survey participants were from Northwest Normal University and provided informed consent online. We distributed recruitment information in student exchange groups and campus forums. The target participants are all students under closed management.^3^ They will be informed of the purpose and results of the study as far as possible. One thousand sixty-two Northwest Normal University students anonymously completed the test. The collected questionnaires were screened according to the response time, and 810 valid questionnaires were included after excluding random and regular questionnaires. The effective rate was 76.27%. A total of 810 participants (*M*_*age*_ = 20.43, *SD* = 1.67, range = 17-30 years, 89.6% female, 31.4% freshman, 25.2% sophomore, 28.0% junior, 9.4% senior, 6.0% graduate student) were included in the final statistical analysis.

### 2.2 Measures

#### 2.2.1 Mindfulness

Mindfulness was measured by the Five-Factor Mindfulness Questionnaire—Short Form (FFMQ- SF) designed by Bare et al. (50) and revised and verified by Deng et al. (51). The revised scale has 20 items divided into five dimensions: observation, description, conscious action, non-judgment, and non-reaction. Each item was scored on a Likert scale ranging from 1 (not at all) to 7 (completely); the higher the score, the higher the mindfulness trait level. This scale is widely used by scholars in China and abroad (52, 53). In this study, the questionnaire’s Cronbach’s α coefficient was 0.71 and each dimension’s Cronbach’s α coefficient was 0.82, 0.83, 0.86, 0.68, and 0.73, respectively. The results of the confirmatory factor analysis show that the structural validity of the questionnaire is acceptable (χ*2/df* = 5.34, CFI = 0.91, TLI = 0.89, RMSEA = 0.07, SRMR = 0.07).

#### 2.2.2 Psychological resilience

Psychological resilience was measured using the Psychological Resilience Scale designed by Connor and Davidson (54) and revised and verified by Yu and Zhang (55). The scale has 25 items covering three dimensions: tenacity, strength, and optimism. Each item was scored on a Likert scale ranging from 1 (never) to 5 (always). The scores for the items are summed to obtain the psychological resilience score: The higher the score, the stronger the psychological resilience. In this study, this scale’s Cronbach’s α coefficient was 0.96, and each dimension’s Cronbach’s α coefficient was 0.95, 0.89, and 0.72, respectively. The results of the confirmatory factor analysis show that the structural validity of the questionnaire is acceptable (χ^2^*/df* = 5.81, CFI = 0.91, TLI = 0.90, RMSEA = 0.08, SRMR = 0.04).

#### 2.2.3 Depression symptoms

Depression symptoms were measured using the Chinese version of the Beck Depression Scale, revised and verified by Wang et al. (56). The scale has 21 items, each with a score between 0 and 3. The total score is the sum of the scores of all items: The higher the score, the more severe the individual’s depression symptoms. The total score on the scale ranged from 0 to 63 points. A score of 0–13 points indicates no depression symptoms, 14–19 points indicates mild depression symptoms, 20–28 points indicates moderate depression symptoms and 29–63 points indicates severe depression symptoms. This scale’s Cronbach’s α coefficient was 0.931 in this study. The results of the confirmatory factor analysis show that the structural validity of the questionnaire is acceptable (χ^2^*/df* = 4.84, CFI = 0.91, TLI = 0.90, RMSEA = 0.07, SRMR = 0.05).

### 2.3 Data analysis

The lavaan R package software and SPSS 24.0 were used to store and manage data. Data on mindfulness, psychological resilience, and depression symptoms are all collected using self-report scales, which may lead to common method bias (57). This study adopted anonymous measurement and reverse scoring methods to control for the common method bias. After data collection, the Harman univariate test was used to measure the size of the common method deviation. Unrotated exploratory factor analysis results extracted a total of 10 factors with eigenvalue roots greater than 1. The maximum factor variance explanation rate was 19.70%, lower than the critical standard of 40%. This indicates there is no evident common method bias in the present study. Descriptive statistics and correlation analyses were performed. The structural equation model and bootstrap test were used to examine the bootstrap method. The 95% upper and lower limits of confidence intervals (CIs) were used to investigate the mediating effect of psychological resilience between mindfulness and depression symptoms.

## 3 Results

### 3.1 Preliminary analysis

A 2 (gender: male, female) × 3 (variables: mindfulness, psychological resilience, depressive symptoms) multivariate ANOVA was conducted. The results showed that the interaction between gender and the variables was significant (*F* = 3.11, *p* < 0.05, η*_*p*_*^2^ = 0.008). Simple effect analysis showed that there was no significant gender difference in mindfulness (*F* = 0.10, *p* > 0.05, η*_*p*_*^2^ = 0.001) and psychological resilience (*F* = 1.96, *p* > 0.05, η*_*p*_*^2^ = 0.000). Furthermore, the gender difference in depressive symptoms was significant (*F* = 5.87, *p* < 0.05, η*_*p*_*^2^ = 0.006). The depressive symptom score for men was significantly higher compared to women (*F* = 4.64, *p* < 0.05, η*_*p*_*^2^ = 0.006).

A 5 (grade: freshman, sophomore, junior, senior, graduate) × 3 (variables: mindfulness, psychological resilience, depressive symptoms) multivariate ANOVA was conducted to test grade differences among mindfulness, psychological resilience, and depression symptoms. The results revealed no significant differences among them (*F* = 1.35, *p* > 0.05, η*_*p*_*^2^ = 0.007). Thus, Hypothesis 1 of this study was partially confirmed. Table 1 shows college students’ scores for mindfulness, psychological resilience, and depressive symptoms according to grade and gender.

**TABLE 1:** Descriptive statistics of grades and gender.

Grades	Gender	PS (*M* ± *SD*)	MD (*M* ± *SD*)	DP (*M* ± *SD*)
First year	Male	91.47 ± 2.87	88.07 ± 1.90	8.63 ± 1.36
	Female	90.79 ± 1.05	88.47 ± 0.70	5.24 ± 0.50
Second year	Male	98.90 ± 3.60	92.00 ± 2.39	2.95 ± 1.70
	Female	90.49 ± 1.16	90.40 ± 0.77	5.20 ± 0.55
Third year	Male	84.75 ± 3.51	85.20 ± 2.33	7.30 ± 1.66
	Female	92.05 ± 1.09	90.63 ± 1.28	5.16 ± 0.52
Last year	Male	90.30 ± 4.97	85.70 ± 3.30	10.70 ± 2.35
	Female	91.20 ± 1.93	90.64 ± 1.28	3.86 ± 0.91
Postgraduate	Male	97.80 ± 7.02	94.00 ± 4.66	4.40 ± 3.32
	Female	85.16 ± 2.37	85.11 ± 1.57	6.82 ± 1.12

*N* = 810, Grade was dummy coded such that 1, first year; 2, second year; 3, third year; 4, fourth year; 5, postgraduate; PS, Psychological resilience; MD, Mindfulness; DP, Depression symptoms.

In this study, the total score obtained on the Beck Depression Scale for college students ranged from 0 to 55; the average score was 5.37 ± 7.47. According to the demarcation line, 706 college students exhibited no depressive symptoms, accounting for 87.2% of the total; 104 college students showed depressive symptoms, accounting for 12.8%. Among them, 59, 32, and 13 college students had mild, moderate, and severe depression symptoms, accounting for 7.3, 4, and 1.6% of the total, respectively. The level of college students’ depression symptoms in this study was significantly lower than that of the college students in the study by Jiang (*t* = -8.28, *p* < 0.001, 7.54 ± 6.31) (58).

### 3.2 Correlation between mindfulness, psychological resilience, and depression symptoms

Table 2 shows the means, standard deviations, and Spearman correlation coefficients for all variables in the current study. Due to depression symptoms having a non-normal distribution, the Spearman correlation test was used to test the correlation coefficient in our study. Correlation analysis showed that during the epidemic prevention and control period, college students’ mindfulness exhibited a significant negative correlation with depression symptoms, a significant positive correlation with psychological resilience, and a significant negative correlation between psychological resilience and depression symptoms. This confirms Hypothesis 2.

**TABLE 2:** Descriptive statistics and Spearman correlations for all measures.

Measure	*M*	*SD*	1	2	3
1 Mindfulness	89.45	10.51	1		
2 Psychological resilience	90.87	15.77	0.65**	1	
3 Depression symptoms	5.37	7.47	−0.36**	−0.39**	1

*N* = 810, Depression scale was dummy coded such that 0, no depression symptoms; 1, mild depression symptoms; 2, moderate depression symptoms; and 3, severe depression symptoms. *p < 0.05, **p < 0.01, ***p < 0.001.

### 3.3 Mediating effect of psychological resilience

During the epidemic prevention and control period, the correlation analysis results showed a significant negative correlation between college students’ mindfulness and depression symptoms and between psychological resilience and depression symptoms. There was also a significant positive correlation with psychological resilience, thus fulfilling the mediation effect analysis conditions. A structural equation model was used to investigate the mediating effect of psychological resilience on mindfulness and depressive symptoms. Mindfulness was an exogenous latent variable, psychological resilience was an endogenous latent variable, and the depression scale was an observational variable as it had only one dimension. Mindfulness includes observation (a1), description (a2), conscious action (a3), non-judgment (a4), and non-reaction (a5) as potential variables. Psychological resilience includes tenacity (b1), strength (b2), and optimism (b3) as potential variables. The measurement model was created using the item parceling method, adjusting the model according to the MI index. The fitting index of the intermediary model was χ*2/df* = 9. 12 (χ*2* = 136. 84, *df* = 15), CFI = 0. 97, TLI = 0. 94, RMSEA = 0. 10, SRMR = 0.04. The maximum likelihood ratio method in SEM causes the chi-square to expand significantly when the number of samples is greater than 500; hence the goodness of fit of the model is acceptable overall.

**FIGURE 1:**
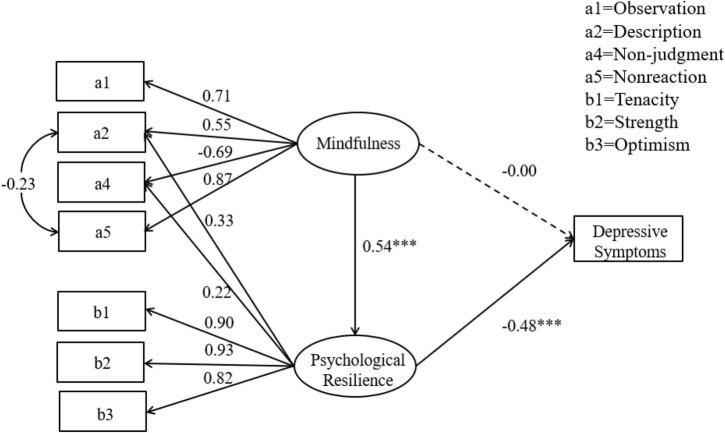
Mediation effect model of psychological resilience between mindfulness and depression symptoms. *p < 0.05, **p < 0.01, ***p < 0.001.

The path coefficients and relationships between the three variables are shown in Figure 1. Mindfulness was significantly positively correlated with psychological resilience (path coefficient is estimated at 0.54). In contrast, psychological resilience was significantly negatively correlated with depression symptoms (path coefficient is estimated at -0.48). Psychological resilience played a complete mediating role between mindfulness and depressive symptoms (the mediating effect is -0.26).

The bootstrap method was used to test the intermediary effect of the data collected in this study. According to Preacher and Hayes (59), the sample size was 5,000. At the 95% confidence interval, the results of the direct effect of mindfulness on depressive symptoms included 0 (LLCI = −0.06, ULCI = 0.06), indicating that the direct effect of mindfulness on depressive symptoms was not significant. The direct effect of mindfulness on psychological resilience did not include zero (LLCI = 0.46, ULCI = 0.72), indicating that the direct effect of mindfulness on psychological resilience was significant. The results of psychological resilience on depression symptoms did not include 0 (LLCI = −0.25, ULCI = −0.17), indicating a significant direct effect on depression symptoms. The result of the mediation effect did not include 0 (LLCI = −0.16, ULCI = −0.09), indicating that the mediation effect of psychological resilience was significant. Therefore, mindfulness is negatively correlated with depression symptoms, and psychological resilience plays a completely intermediary role, confirming Hypothesis 3.

### 3.4 Path analysis of the mediation model

We analyze the factor load of each potential variable. The factor load of mindfulness in the model was more than 0.50, and the factor load of non-reaction was approximately 0.90. This indicates that observation, description, non-judgment, and non-reaction are typical dimensions of mindfulness, and individuals can refrain from reacting to perceived cognition and emotion. This plays an important role in the relationship between mindfulness and depressive symptoms. The factor loads of psychological resilience were all above 0.80. Moreover, tenacity and strength factor loads were above 0.90, indicating that tenacity, strength, and optimism are typical dimensions of psychological resilience. Mindfulness is involved in improving the strength and tenacity of college students in the epidemic environment. In addition, the dimension of conscious action (path coefficient is estimated at 0.06) was not significant to the total score of mindfulness (*p* > 0.05, LLCI = −0.29, ULCI = 0.16). As in previous research (60), this dimension was deleted from the model. The conscious action package did not perform well in the model. In the epidemic context, cognitive awareness and acceptance of inner experience are more important than focusing on the current action. In addition, the description and non-judgment dimensions of mindfulness have cross-loadings on psychological resilience, which was notable. Many researchers pointed out that when CFA involves personality, the cross-loadings between factors may reflect the extensive relationship between personality rather than the measurement problem; therefore, its theoretical significance should be considered (61, 62). Joshanloo et al. (63) believe that the cross-loadings close to or greater than 0.3 is an important factor in the structure. We modified the model according to the MI index. The results showed that the descriptive (path coefficient is estimated at 0.55) and non-judgment (path coefficient is estimated at 0.69) dimensions still indicated a higher load on the mindfulness factor, with psychological resilience cross-loadings of 0.22 and 0.33, respectively. Further analysis showed that both descriptive (*p* < 0.000, LLCI = 0.29, ULCI = 0.51) and non-judgment dimensions (*p* < 0.000, LLCI = 0.15, ULCI = 0.37) had significant effects on the psychological resilience path. In the epidemic context, this result indicates that college students can clearly perceive their inner experiences and accept them without judgment. This process has a particular theoretical relationship with the psychological resilience mechanism.

## 4 Discussion

This study explored the internal mechanisms of mindfulness on depression symptoms from the perspective of psychological resilience. The results showed that college students’ psychological resilience played an intermediary role between mindfulness and depressive symptoms. This result verified the protective role of mindfulness and psychological resilience in the general population. They also solved the existing problem of lack of sample population representativeness in previous studies. The results of this study bolster existing literature on the depression status of college students in the context of closed management and COVID-19. The results can be leveraged to create and implement targeted interventions to improve mental health.

No significant differences were detected in mindfulness, psychological resilience, and depression symptoms among college students of different grades. This may be because the psychological development of college students of all grades is focused on the early adulthood stage. Thus, their psychological development levels are similar. No significant gender differences were found in mindfulness and psychological resilience. Men’s depression levels were higher than women’s, which contradicts previous studies; early epidemic research indicates that young women are at a higher risk of mental health problems (49). Women have been found to have higher levels of depression and lower levels of psychological resilience than men (7, 48). Women’s sensitivity to perceived pressure is bound to worsen their mental health due to anxiety about the epidemic and isolation. However, with the public’s increased understanding and adaptation to the epidemic, its impact on women’s mental health has gradually decreased. Recent studies have shown no significant gender differences in mindfulness or psychological resilience, consistent with the results of this study (64, 65). In addition, higher levels of depression symptoms among men in this study may be related to the more considerable adaptive pressure faced by male students. In Chinese culture, boys and men are less likely to actively seek social support (66), resulting in greater adaptive pressure. In addition, sports play a more obvious role in promoting boys’ mental health (67), and the inability to carry out outdoor sports in an isolated environment may cause more psychological discomfort. This suggests that colleges and universities should actively focus on the impact of closed management on the mental health of male students. It should be noted that the effect of this result is small and should be verified using a larger sample or a more rigorous experimental design.

This study found that the depression symptom level of college students during the closed management period was slightly lower than under normal social conditions (58), which differs from previous studies. Previous studies have pointed out that the COVID-19 epidemic and its associated feelings of isolation have increased people’s psychological problems to varying degrees. Isolated personnel have a high level of stress and different levels of depression symptoms, anxiety, and other emotions. The detection rate of depression has increased significantly since the epidemic outbreak (68, 69). This difference may be due to the increased publicity of the epidemic’s effects by the government, schools, and health experts; China normalized the prevention and control of the epidemic, and college students obtained a better understanding of these measures (70, 71). This stabilizes the students’ overall mood when facing closed management situations and enables them to adopt a positive self-regulation mode (72, 73). At the same time, repeated waves of infection have also created opportunities for college students to adapt after trauma (74). Their psychological resilience and mindfulness have played a positive role in their protection, and they can cope better with the epidemic’s impact. Moreover, colleges and universities attach substantial importance to their students’ mental health. These institutions are actively implementing a series of targeted measures during the epidemic prevention and control period. These measures may include conducting online individual and group psychological counseling, establishing a psychological hotline, introducing communication channels to understand student demands, gradually opening public areas based on the infection rates, and organizing sports competitions and physical exercise punch-in activities. All such measures can alleviate college students’ psychological pressure during isolation. These measures significantly contribute to relieving depression symptoms and promoting mental health among college students (75, 76). On the premise that the isolation measures are reasonable and scientific, the isolated will feel that they have been better protected, thereby reducing negative emotions and stress and improving their overall mental health.

The study found that college students’ mindfulness was directly and negatively associated with individual depression symptoms during the closed management period. The higher the level of mindfulness, the fewer the depressive symptoms; this is consistent with previous research results (18, 19). As an important protective factor for mental health development, mindfulness significantly contributes to relieving depression and other negative emotions (11, 77). Tran et al. (78) believed that the most important mechanisms of mindfulness are decentering and non-attachment. “Decentering” means that individuals can shift their attention away from negative cognition and emotions and stop themselves from falling into a cycle of negative emotions. “Non-attachment” means accepting and not indulging in the inner experience. When an individual is worried about the epidemic or the future, mindfulness acts on attention distribution through decentering, making the individual focus on awareness and reducing the automatic response to negative emotions (79). Individuals with a high level of mindfulness are better at observing their surrounding environment outwards and identifying their inner experience inwards, which helps eliminate the persistent negative impact of the epidemic. When individuals can accept and not judge their inner emotions and ideas, it is helpful for them to actively self-regulate, block negative thinking, and prevent invasive rumination (80, 81). This can promote objective recognition of their own state, distinguish between “ideas” and “reality,” and disassociate from the negative thinking mode (82–84). Acceptance and non-judgment are the core elements of mindfulness that improve emotions states (85, 86). This mode of thinking helps individuals maintain high mental health in an epidemic-pressured environment. In this study’s model, the observation, description, non-judgment, and non-reaction dimensions of mindfulness have a higher degree of fit, which verifies the above mechanism. The conscious action dimension of mindfulness did not fit well in this model, which may be related to the multitrait pattern of mindfulness. The feature structure of mindfulness is not unidimensional; there are widely distributed and different mindfulness feature groups in the population (87, 88). The five dimensions—observation, description, conscious action, non-judgment, and non-reaction—have different levels in different mindfulness trait groups (89). Research also shows that mindfulness’ conscious action is not significantly associated with long-term depressive symptoms (60). It is also possible that college students’ actions are restricted to a certain extent in an isolated environment, and their conscious actions are, therefore, poorer (90).

Research has demonstrated that psychological resilience completely mediates mindfulness and depression symptoms; mindfulness can reduce the depressive symptoms of individuals by improving their psychological resilience. All dimensions of the model’s psychological resilience played a significant role. The tenacity and strength of psychological resilience were slightly stronger than those of optimism. In other words, mindfulness has strengthened individual resources and tolerance for epidemics. According to previous studies, mindfulness can reduce automatic reactions, redundant thinking, rumination, and avoidance thinking by acknowledging one’s thoughts, feelings, and evaluations. Simultaneously, psychological resilience can help cope with stressful environments by mobilizing psychological resources, increasing positive emotions, and achieving good adaptation. This is a process of recognizing and accepting emotions and improving mental health through emotion regulation strategies, consistent with mindfulness mechanisms (43). It also verifies Kumpfer’s resilience framework, that is, psychological resilience mediates the impact of individual characteristics on adaptive results (30). The anterior radius of the mediation model shows that mindfulness is positively correlated with psychological resilience, consistent with previous studies (36). When individuals perceive and recognize the negative impact of an epidemic, they can improve their attention strategies through decentering to promote the development of psychological resilience. The attitude of non-attachment improves the individual’s positive cognitive ability through acceptance and non-judgment (91), thereby improving their psychological resilience (92). The posterior radius of mediation shows that college students’ psychological resilience is negatively correlated with their depressive symptoms, consistent with the findings of previous studies (93). People with high psychological resilience have more positive psychological traits. Thus, they are more resilient, powerful, and optimistic. They can overcome difficulties, have positive expectations for the future and the present, can focus more on positive experiences during the epidemic and closed management, actively adjust their emotional state and reduce negative emotional experiences, such as depression and anxiety (94, 95). In the context of an epidemic, a relatively monotonous living environment tests individuals’ psychological resilience. The epidemic has resulted in several challenges, such as employment concerns and increased educational pressure. Highly psychologically resilient individuals use positive emotion regulation strategies, block the automatic response to risk factors, and actively and flexibly mobilize internal and external resources to cope with these pressures to avoid succumbing to negative emotions (32, 96).

The model’s description and non-judgment dimensions of mindfulness have the relevant cross-loadings on psychological resilience. This may be related to similarities in psychological resilience and mindfulness mechanisms of action. First, mindfulness is based on the development of cognitive functions. Individual working memory and cognitive inhibition, in particular, are highly correlated with descriptive and non-judgmental levels (97). Furthermore, cognition is an important pathway for resilience. Secondly, the descriptive dimension of mindfulness describes the observed experience with words, thereby reducing automatic and inappropriate responses. Non-judgment means adopting a non-evaluative attitude toward the experience. This helps reduce reactions to negative emotions and greatly reduces the pain caused by secondary reactions (98). Psychological resilience emphasizes using positive emotional regulation strategies to avoid automatic responses to negative emotions. Psychological resilience is highly similar to the descriptive and non-judgmental mechanisms of action. In addition, Burzler et al.’s research pointed out that the effect of mindfulness on depressive symptoms is primarily carried out by identifying emotions, accepting emotions, and adopting positive emotion regulation strategies (99). This provides another perspective on the cross-loading of the description and non-judgmental dimensions of resilience and mindfulness in the model. Future research can build non-judgmental, observational, and resilience models to further validate this mechanism through mindfulness intervention experiments.

In summary, mindfulness and psychological resilience should not be limited to clinical groups. Regular college students can also prevent external shocks by improving their mindfulness and psychological resilience. In the epidemic context, mindfulness and psychological resilience are important resources for the general population to maintain mental health. We should actively promote its protective role in minimizing the epidemic’s impact on mental health. The mediation model shows that colleges and universities can introduce group counseling courses based on psychological resilience and mindfulness during epidemic prevention and control periods. Furthermore, the popularization of relevant psychological knowledge through online and offline psychological counseling and mental health lectures can help minimize the effects of depression. It is also necessary to nurture the internal psychological resources of college students through interventions such as meditation training and mindfulness intervention. This, in turn, will improve their psychological resilience, increase their ability to deal with stressful events, and improve their mental health. These measures are necessary to protect the students’ mental health and reduce the risk of depression.

## 5 Limitations and future research directions

First, the study sample was from regular universities in China, with a high proportion of female students, which affects the generalization of the conclusions to a certain extent. Second, the study used self-reported data, which may have introduced recall and social desirability biases. Third, this study adopted a cross-sectional design, which cannot draw a causal inference between mindfulness and depression symptoms. Further studies can adopt a longitudinal research design to draw causal inferences and use more objective experimental methods to verify the protective mechanism of mindfulness and psychological resilience on depressive symptoms. Taking college students during the closed management period as the research object and setting up a control experiment of mindfulness intervention to explore the development trend of mindfulness, psychological resilience, and depression symptoms, so as to verify the protective effect of mindfulness and psychological resilience on mental health in the COVID-19 situation.

## 6 Conclusion

This study discusses the relationship between mindfulness, psychological resilience, and depressive symptoms within the context of COVID-19. Moreover, the study verifies the intermediary role of psychological resilience between mindfulness and depressive symptoms. The study found that the depression level of male students was slightly higher than female students under the closed management system. College students’ mindfulness level was negatively associated with depression symptoms and positively associated with psychological resilience; thus, psychological resilience was negatively associated with depression symptoms and completely mediated the effect of mindfulness on depression symptoms. Colleges and universities can improve the mental health of college students affected by the epidemic through mindfulness interventions and meditation training.

## Data availability statement

The raw data supporting the conclusions of this article will be made available by the authors, without undue reservation.

## Author contributions

YJ designed the study and supervised the project. YJ and YY helped revise the manuscript. YY and YH analyzed the data. ZY drafted the manuscript and collected the data. FL and HM distributed questionnaires and screened participants. All authors have read and approved the final manuscript.

## Footnotes

1 “Normalization” refers to the gradual transformation of affairs or states that originally had sudden, uncertain, and accidental characteristics into daily, regular, and lasting ones. Normalization of epidemic prevention and control means that due to the rapid spread of COVID-19 around the world, China may coexist with the epidemic indefinitely. In view of the epidemic development worldwide, the Chinese government has transformed emergency measures taken under certain situations into sustainable and long-term prevention and control measures (2).

2 “Clearing” is the process of clearing the epidemic. “Dynamic” means that the occurrence and elimination of the epidemic are constantly changing. Dynamic zeroing means that the Chinese government does not seek to eliminate all domestic epidemics. Instead, it quickly identifies and handles the COVID-19 epidemic, cuts off the transmission chain of the virus, and controls it in a relatively short period of time. This prevents the spread of novel coronavirus virus in the place where the epidemic originates, as well as other regions. As a result, Chinese society as a whole is gradually moving toward the elimination of COVID-19 (3).

3 Closed management is a common management measure adopted by Chinese Universities during the outbreak of COVID-19. It mainly refers to the temporary and strict closed management system adopted to prevent the spread of the virus. Specifically, teachers and students are prohibited from moving across campuses. Teachers must isolate at home and students in their dormitories. Only online teaching takes place. Students are not allowed to enter or leave the school, and most of the time remain isolated in the dormitory. They can only move within the limited space on campus within specified time frames. Libraries, study rooms, stadiums and other public places are temporarily closed. Students go to the canteen to order food at a specified time and take it back to the dormitory to eat. Express delivery and takeaways are not permitted on campus.
